# Comparative effectiveness of 12-week open- and closed-skill exercises on executive function in children with ADHD and developmental dyslexia: a randomized controlled trial

**DOI:** 10.1186/s12887-026-07105-w

**Published:** 2026-06-15

**Authors:** Tianshen Quan, Jiefeng Ye, Qian Yu, Feilong Zhu, Lin Zhang

**Affiliations:** 1 Physical Education School, Shenzhen University, Shenzhen, China; 2 Shenzhen Senior High School, Shenzhen, China; 3 Shenzhen Bao’an District Shangxing School, Shenzhen, China; 4 Beijing Sport University, Beijing, China

**Keywords:** ADHD + DD, Table tennis training program, Motor-based intervention, Executive function

## Abstract

**Background:**

Children with attention deficit/hyperactivity disorder and comorbid developmental dyslexia (ADHD + DD) demonstrate both core symptoms of ADHD and pronounced reading difficulties, which further compromise executive function (EF). This study compared the effects of open-versus closed-skill exercises (OSE vs. CSE) on EF in children with ADHD + DD.

**Methods:**

Thirty children (6.9–8.5 years) with ADHD + DD were randomly assigned to OSE (*n* = 15) or CSE (*n* = 15) training, with 15 typically developing (TD) children receiving CSE. All completed 12-week, thrice-weekly, moderate-to-high intensity (> 60% VO₂ max) sessions. OSE performed table tennis; CSE did track-and-field. EF was assessed with SCWT, CFT, and TMT; visual perception used DTVP-3. Data were analyzed by two-way repeated-measures ANOVA, with ANCOVA used to adjust baseline differences.

**Results:**

Compared with TD peers, ADHD + DD children exhibited impairments in inhibitory control (including Stroop A/B/C/D RT, Stroop B/D error count, and word interference time), all working memory indices, cognitive flexibility (digit-alphabet linking time), and all visual perception measures (all *p* < 0.05). After intervention the outcomes revealed significant Group × Time differences in EF, including inhibitory control (Stroop B/D RT, word interference time), working memory (delayed detail and structural memory), and cognitive flexibility (digit–alphabet linking time) (*p* < 0.05). Post-hoc tests confirmed that OSE yielded significantly greater improvements than CSE. For secondary visual perception outcomes, all measures showed significant Group × Time interactions (all *p* < 0.05). Further adjusted analyses demonstrated that OSE outperformed CSE significantly in visual-motor integration (VMI), copying ability, motor-reduced visual perception (MRP), form consistency, and general visual perception (GVP) (all *p* < 0.05).

**Conclusions:**

OSE (table tennis training) has superior efficacy in improving EF and visual perception in children with ADHD + DD compared with CSE (track-and-field training), providing preliminary empirical evidence for a feasible non-pharmacological intervention strategy for this population.

**Supplementary Information:**

The online version contains supplementary material available at 10.1186/s12887-026-07105-w.

## Background

Attention-deficit hyperactivity disorder (ADHD) is a prevalent neurodevelopmental disorder in childhood, characterized by inattention, hyperactivity, and impulsivity [1, 2]. These symptoms impair academic performance, social adaptation, and interpersonal relationships in school-aged children, and increase the risk of antisocial behavior, criminality, and substance abuse later in life [3–5]. Research suggests that approximately 65% of children with ADHD present with at least one comorbid psychiatric disorder, with learning disabilities (LD) being among the most prevalent [6, 7]. Within the LD population, nearly 80% are diagnosed with developmental dyslexia (DD), which is marked by poor reading skills, slow handwriting, and frequent spelling errors [8, 9]. Notably, an estimated 25% – 40% of children with ADHD also have DD, and conversely, 25% – 40% of children with DD have ADHD, a condition associated with pronounced reading difficulties, emotional disturbances, behavioral problems, and deficits in social adaptability [10]. This dual diagnosis further exacerbates core cognitive and behavioral impairments, particularly deficits in multiple components of executive function (EF), placing affected children at a greater disadvantage relative to their typically developing peers [11].

For children with ADHD + DD, clinical interventions typically involve pharmacological treatments to alleviate core ADHD symptoms, often supplemented with learning skills training and cognitive-behavioral therapy (CBT) [12, 13]. In recent years, however, physical activity interventions have gained increasing attention as a non-pharmacological approach for managing ADHD-related impairments. Evidence suggests that diverse forms of physical activity, including open- skill exercises (OSE) and closed-skill exercises (CSE), can positively impact cognitive function, emotional regulation, and obesity-related characteristics in children with ADHD by modulating intensity and duration [14–16]. Nonetheless, the comparative efficacy of different exercise modalities remains inconclusive, highlighting the need for further research to determine optimal intervention strategies.

Among various forms of physical activity, racket sports such as table tennis are thought to confer distinct cognitive benefits due to their high demands on sustained attention, eye – hand coordination, and EF [17]. These activities require continuous concentration, rapid responses, and precise tracking of ball trajectories, making them particularly suitable for enhancing attentional control and motor skills in children with ADHD + DD. Accordingly, this study aimed to address the specific developmental needs of children with ADHD + DD by comparing the effects of table tennis (OSE) versus track-and-field (CSE) on their EF. The ultimate objective was to establish a scientifically validated physical activity intervention and to provide theoretical and practical guidance for primary school physical education instructors in designing tailored training programs for affected children.

## Methods

### Study design

This study adopted a parallel controlled design, with an exercise intervention period lasting 12 weeks. Pre-testing was conducted one week prior to the intervention. The assessment battery included three computerized tests measuring core components of EF—the Stroop Color-Word Test (SCWT), Category Fluency Test (CFT), and Trail Making Test (TMT)—all administered via E-Prime, which were designated as primary outcomes, together with the Developmental Test of Visual Perception-Third Edition (DTVP-3) as a secondary outcome. All data collection sessions were uniformly scheduled between 7:00 and 9:00 a.m., with subjects completing the assessments in a quiet, enclosed environment. Post-testing was carried out within one week after the exercise intervention concluded, under identical environmental conditions and testing protocols as the pre-test.

### Participants

A total of 30 children aged 6.9 to 8.5 years diagnosed with ADHD + DD by a psychiatrist, based on the Diagnostic and Statistical Manual of Mental Disorders, Fifth Edition (DSM-V) [18] and 15 typically developing (TD) children were enrolled into this randomized controlled trial (ChiCTR2200065413) from a public primary school in Beijing, China. The inclusion criteria were as follows: (a) Diagnosis of ADHD according to DSM-V criteria; (b) Diagnosis of Dyslexia according to DSM-V criteria; (c) A Wechsler Intelligence Scale for Children (WISC) score ≥ 90, ensuring exclusion of children with severe neurodevelopmental or psychiatric disorders. In accordance with the requirements of the local ethics committee, both the participants and their legal guardians were provided with detailed information documents and subsequently signed informed consent forms.

**Fig. 1:**
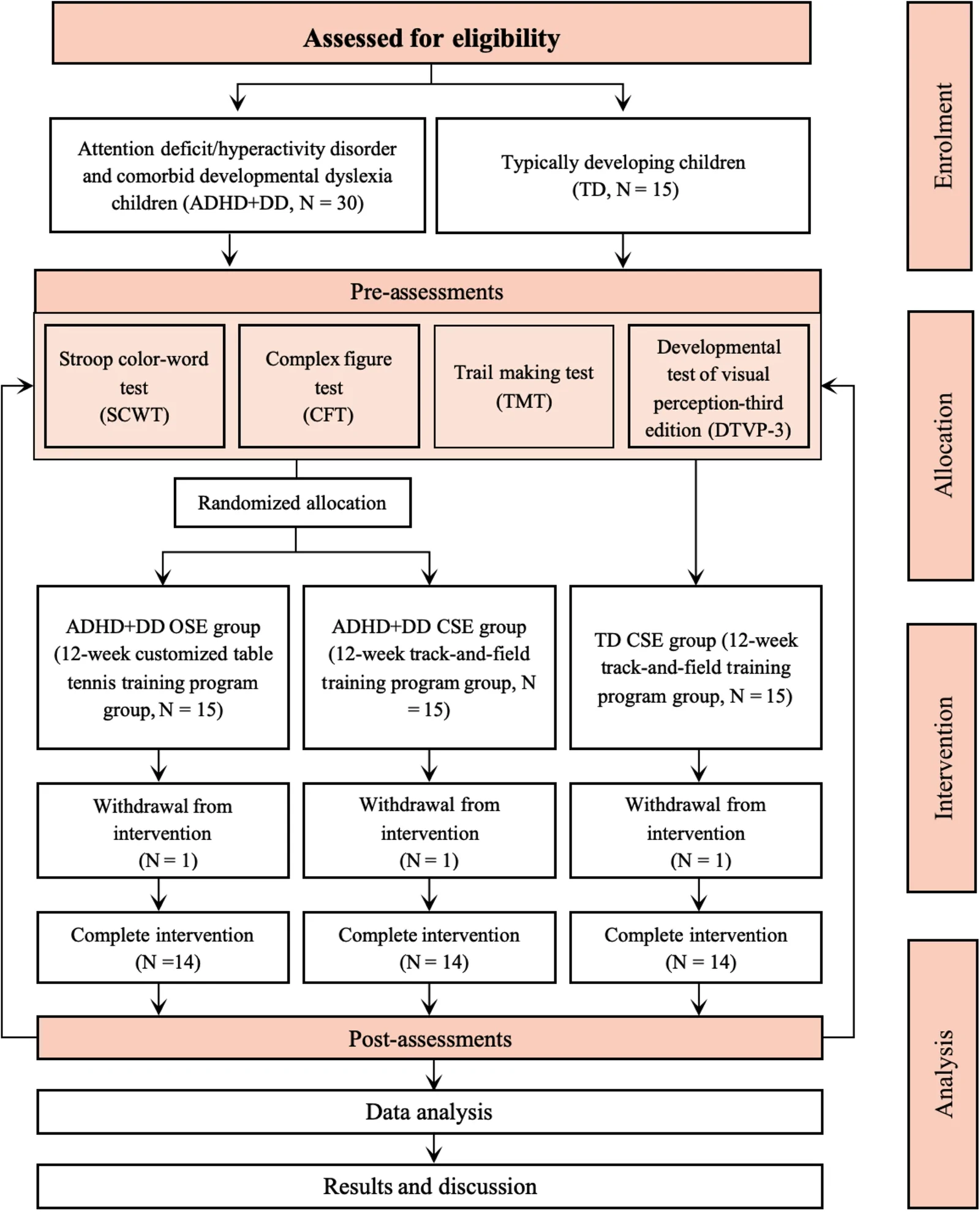
Participant selection flowchart of the study

The 30 children with ADHD + DD were randomly assigned into two groups: an OSE group (ADHD + DD OSE, *n* = 15, mean age 7.8 ± 0.7 years) and a CSE group (ADHD + DD CSE, *n* = 15, mean age 7.7 ± 0.85 years). TD children were recruited to undergo CSE activities (TD CSE, *n* = 15, mean age 7.8 ± 0.61 years) (Fig. 1). This study utilized simple randomization with SPSS-generated allocation schedules by personnel uninvolved in recruitment. Participants and data analysts remained blinded to group assignment. Independent researchers, unaware of group allocations, conducted both separate intervention sessions and data analysis. The protocol was approved by the local ethics committee following the Declaration of Helsinki. Attrition occurred in each group.

### Study interventions

The OSE (table tennis) and CSE (track-and-field) programs were conducted three sessions per week for 45 min per session, over a 12-week period. Each session included an 8-minute warm-up/cool-down (55–60% max heart rate). The warm-up comprised: 5-minute jogging, 2-minute dynamic stretching, and 1-minute acceleration runs. Training intensity was required to be maintained above 60% of maximum heart rate.

Based on Newell’s Motor Development Model and expert consultations, the OSE (table tennis) program targets motor development, EF (inhibitory control, working memory, cognitive flexibility), and visual perception deficits in children with ADHD + DD through task constraints and environmental design. Task constraints: task difficulty was dynamically adjusted based on individual ability levels by manipulating parameters such as speed, color, direction, and complexity. Environmental design: Variations in surface texture, markers, and obstacles were used to enhance motor adaptability and perceptual processing (Table 1).

**Table 1:** Exercise protocol

Process		Training content	
		Table tennis training	Track-and-field training
Fundamental Stage	1-2 weeks	Goal: Cognition of table tennis.1. Grip techniques (Penhold, shake hand).2. Forehand/backhand swing mechanics.3. Footwork drills (side shuffle, crossover steps).4. Fun games like "Ping-Pong Bowling" and "Rolling Ball Relay".	Goal: Cognition of core movements.1. Marching in place transitioning to high knees.2. Standing long jump with distance markers.3. Seated forward soft ball push.4. Fun games like: "Animal Imitation Exercises" and "Rhythm Clapping Game".
	3-4 weeks	Goal: inhibitory control, cognitive flexibility, visuospatial ability, and visual-motor integration.1. Single hand toss and catch.2. Alternating hands toss and catch.3. Single hand toss and grab.4. Alternating hands toss and grab.5. Bounce and catch.6. "Ball-in-the-box" precision drill.7. "Cup-and-ball" dribbling exercises.	Goal: Strengthening of movement rhythm.1. Rhythmic high knee march.2. 3-step approach single-leg hop.3. Kneeling ball toss & catch.4. Elastic band resistance swings.5. Fun games like: "Listen & Act".
	5-6 weeks	Goal: inhibitory control, cognitive flexibility, working memory capacity, eye-hand coordination, and visual constancy.1. Fundamental paddle balancing drills.2. Dynamic balancing with progressive difficulty: (Basic moving drills, Advanced obstacle navigation and Auditory-cued stationary/moving transitions).3. Fun skill games: "Pole-assisted catches"; " Team balancing relays "; " Alternative tool exercises (spoons, chopsticks)".	Goal: Expansion of movement range.1. Vertical jump with reach.2. Continuous frog jumps.3. Standing chest push throw.4. Fun games like: "Frog Jump Championship", "Kangaroo Reach Challenge" and "Bear Throw Tournament".
Development Stage	7-8 weeks	Goal: inhibitory control, cognitive flexibility, visual discrimination, and visuospatial ability.1. Stationary ball bouncing drill.2. Wall rally practice.3. Moving ball bouncing drill.4. Alternating forehand-backhand bouncing.5. Partner toss-and-catch exercise.6. "Ball bounce challenge course".	Goal: Foundation of linear speed.1. 30-meter straight sprint.2. Alternate-leg hurdle hops.3. Two-hand overhead ball throw.4. Agility ladder fundamentals (single-foot in, two-foot jump).5. Fun games like: "Traffic Light".
	9-10 weeks	Goal: inhibitory control, visual tracking, visual constancy, eye-hand coordination, and visuo-motor integration.1. Service training drill.2. Forehand/backhand flat serve.3. Backhand push. 4. Forehand drive.5. Continuous rally drill.6. Sloped return training.	Goal：Kinetic chain development drills 1. 20-Meter back-kick run.2. Standing triple jump.3. Medicine ball side throw4. Resistance dynamic exercises (lateral band walk, standing band row).5. Fun games like: "Turn & Catch".
Consolidation Stage	11-12 weeks	Goal: enhance working memory, inhibitory control, cognitive flexibility, and visual discrimination ability.1. Multicolor reaction drill (3-ball random feed).2. Color-selective filtering (develop focus).3. Shuffle-step combos with (Standard FH/BH sequence, Color-assigned strokes).4. Progressive difficulty (Stage 1: Slow feed, 2 colors, Stage 2: Fast random, 3 colors and footwork).	Goal: Application of movement combinations.1. Run-jump combination drill.2. Obstacle course run.3. Throw-catch integration.4. Team game: "Relay pass challenge".

CSE (Track-and-Field) program used jumps, throws, rhythm, and ladder drills to build explosive power and coordination. Its systematic approach progresses from motor cognition to rhythm, spatial adaptation, and force transmission, culminating in skill application and closed-loop learning (Table 1).

### Study outcomes

### Primary outcomes: executive function

EF encompasses three core domains: inhibitory control (assessed by the SCWT), working memory (assessed by the CFT), and cognitive flexibility (assessed by the TMT).

**Fig. 2:**
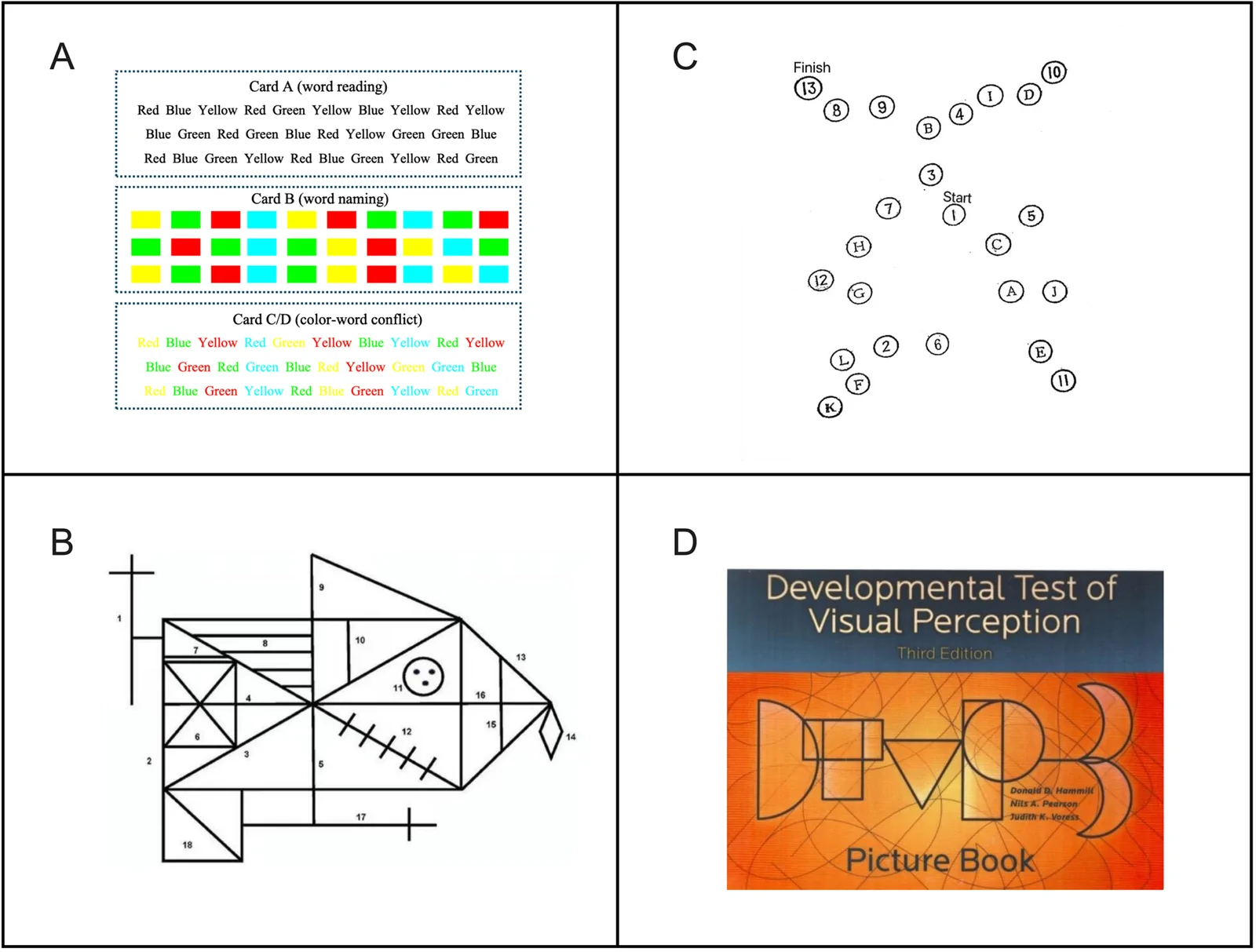
Flow diagram of the executive functional and visual perception tasks. **A** Flow diagram of the stroop color-word test (SCWT); (**B**) Flow diagram of the complex figure test (CFT); (**C**) Flow diagram of the trail making test (TMT); (**D**) Flow diagram of the developmental test of visual perception-third edition (DTVP-3)

Stroop color-word test (SCWT). The test consists of four card sets (A, B, C, D), completed in four steps: (1) Card A: 30 words in four colors (yellow, red, blue, green) printed in matching ink colors (e.g., “red” in red ink). The task is to read the words quickly and accurately. (2) Card B: 30 solid color patches (yellow, red, blue, green). The task is to name the colors quickly and accurately. (3) Card C: 30 color words printed in incongruent ink colors (e.g., “red” in blue ink). The task is to read the word meanings while ignoring the colors. (4) Card D: Same as Card C, but the task is to name the ink colors while ignoring the word meanings. Measured variables include: (1) completion time and error count for each card; (2) color interference time (Card C time – Card A time), reflecting the interference of colors on word reading; and (3) word interference time (Card D time – Card B time), reflecting the interference of word meanings on color naming. Shorter completion times and fewer errors indicate better inhibitory control (Fig. 2A).

Complex figure test (CFT). The Rey-Osterrieth Complex Figure Test requires children to observe the Rey complex figure for 30 seconds, then immediately recall and draw it from memory, followed by another recall after 30 min. Scoring is based on structural and detail features of the figure. Structural scores range from 0 to 6 points, and detail scores range from 0 to 36 points. Four dimensions are derived: immediate structure, immediate details, delayed structure, and delayed details. Higher scores reflect better memory ability (Fig. 2B).

Trail making test (TMT). The TMT consists of two parts. Part A (Digit connection): Numbers 1 to 25 are randomly scattered on the page. The participant must connect them in ascending order as quickly as possible. Completion time and error count are recorded. Part B (Digit-alphabet linking time): Numbers 1 to 13 and letters A to L are randomly scattered. The participant must alternate between numbers and letters in the sequence 1-A-2-B-3-C-… while maintaining speed and accuracy. Shorter completion times and fewer errors indicate better cognitive flexibility, whereas longer completion times and more errors indicate poorer performance. Part A primarily evaluates processing speed and visual scanning, whereas Part B reflects task-switching ability and executive function (Fig. 2C).

#### Secondary outcomes: visual perceptual ability

The Developmental Test of Visual Perception–Third Edition (DTVP-3) was used to assess children’s visual perception and visual–motor integration abilities [19]. This scale is standardized for children aged 4 to 12 years and 11 months, with adequate normative validity across race, gender, and handedness [20]. The test procedure was conducted following standardized guidelines (Fig. 2D).The assessment included: Step 1: Calculate raw scores (obtain raw scores for each subtest): (1) Eye-hand coordination – child draws lines/curves within visual boundaries. (2) Copying – child reproduces simple to complex figures. (3) Figure-ground – child identifies hidden numbers in a cluttered background. (4) Visual closure – child selects the correct complete shape from fragmented options. (5) Form consistency – child recognizes target shapes despite scaling, rotation, or shading changes. Step 2: Convert raw scores to standard scores: use the manual’s conversion tables to transform raw scores into standard scores based on the child’s age. Step 3: Compute composite raw scores: (1) Visual-motor integration (VMI) = eye-hand coordination standard score + copying standard score. (2) Motor-reduced visual perception (MRP) = figure-ground + visual closure + form consistency standard scores. (3) General visual perception (GVP) = VMI raw score + MRP raw score. Step 4: Convert composite raw scores to standard scores. Higher scores indicate stronger abilities.

#### Quality monitoring

Before the intervention, tests on EF and visual perception development levels were conducted. To ensure the quality of each indicator in the tests, the intra-group consistency of members was checked prior to the formal tests. The group collectively organized test learning and assessments, followed by repeated evaluations and practice sessions until the consistency of scoring among group members reached over 80%, thereby guaranteeing the accuracy of the test results. All tests were administered by one person, scored by two raters, and finalized by the lead tester to minimize scoring errors.

We used smart fitness trackers to monitor average heart rates per session, ensuring data validity. The class time and attendance frequency for each group were strictly controlled to ensure the complete implementation and progression of the intervention content, while also meeting the total duration requirement of the intervention. Children who did not participate in any of the three training sessions were regarded as having withdrawn from the intervention. During the classes, the children’s performance was monitored and recorded. Based on the observation records and interactions with parents, the intervention program was continuously optimized to ensure the functionality, relevance, and effectiveness of the online sports intervention.

### Statistical analysis

Sample size was estimated using G*Power 3.1 based on a previous study (η² = 0.35, f = 0.73) [21], with α = 0.05 (two-tailed) and power = 0.80, requiring 30 participants (10 per group) for a 3 × 2 repeated-measures design, which was met. Normality and homogeneity of variance were assessed using Shapiro-Wilk and Levene’s tests. One-way ANOVA with Bonferroni post-hoc tests were used for baseline comparisons when assumptions were met; otherwise, Welch’s ANOVA or Kruskal-Wallis H tests with Games-Howell or Dunn’s post-hoc tests were applied. Baseline comparisons were descriptive to assess randomization success; no correction for multiple comparisons was applied, and baseline differences were adjusted using ANCOVA. Primary outcomes were pre-specified as 18 executive function measures (SCWT, CFT, and TMT); secondary outcomes were DTVP-3 visual perception measures. Intervention effects were analyzed using a 3 (group) × 2 (time) repeated-measures ANOVA, with Greenhouse-Geisser correction for violations of sphericity. Effect sizes are reported as η²p. Given the large number of primary outcomes, the Benjamini-Hochberg false discovery rate (FDR) procedure (q = 0.05) was applied to the p-values for the 18 Group × Time interactions, with corrected *p* < 0.05 considered significant (indicated by †). For outcomes with significant interactions or significant group main effects in ANCOVA, post-hoc pairwise comparisons were performed using Bonferroni correction (α = 0.0167). For variables with baseline differences, mixed-model ANCOVA with baseline scores as covariates was used to confirm robustness. Secondary outcomes are reported with unadjusted p-values and interpreted as exploratory. All tests were two-tailed, and data were analyzed using SPSS 27.3 and GraphPad Prism 10.3.

## Results

An average of one participant per group withdrew midway due to personal reasons such as physical discomfort and schedule conflicts, with 14 participants in each group ultimately included in the post-intervention statistical analysis.

### Baseline group comparisons

Baseline EF and visual perception measures were compared among the three groups (TD CSE, ADHD + DD CSE, ADHD + DD OSE; Table 2). The TD group exhibited significantly better performance than the two ADHD + DD groups across inhibitory control (including Stroop A RT, Stroop B RT, Stroop C RT, Stroop D RT, Stroop B error count, Stroop D error count, and word interference time), all working memory indices, cognitive flexibility (digit-alphabet linking time), and all visual perception measures (all *p* < 0.05), with moderate-to-large effect sizes (η² = 0.132–0.669). The two ADHD + DD groups did not differ significantly at baseline (all *p* > 0.05), confirming successful randomization.

**Table 2:** Baseline comparisons of executive function and visual perception among three groups

Variables		Groups			Statistic (χ²/F)	P-value	η²	95% CI	
		TD CSE	ADHD+DD CSE	ADHD+DD OSE					
Executive function (Primary Outcomes)	Inhibitory control capacity								
	Stroop A RT (s)	19.14 ± 3.39	25.43 ± 7.21 #	23.86 ± 2.93 #	9.07	0.001**	0.242	0.030	0.420
	Stroop A error count	0.07 ± 0.27	0.21 ± 0.43	0.21 ± 0.43	1.34	0.512			
	Stroop B RT (s)	24.57 ± 4.07	34.29 ± 7.16 #	32.07 ± 6.74 #	9.61	<0.000***	0.330	0.087	0.498
	Stroop B error count	0.14 ± 0.36	0.86 ± 1.03 #	0.86 ± 0.86 #	6.69	0.035*			
	Stroop C RT (s)	23.07 ± 6.03	35.64 ± 12.29 #	33.50 ± 10.40 #	8.89	0.001**	0.248	0.033	0.425
	Stroop C error count	0.29 ± 0.47	1.14 ± 1.29	1.07 ± 1.07	5.51	0.064			
	Stroop D RT (s)	42.36 ± 7.36	60.64 ± 13.56 #	60.93 ± 12.60 #	11.99	<0.000***	0.381	0.128	0.541
	Stroop D error count	1.00 ± 1.18	2.36 ± 1.15 #	2.64 ± 1.65 #	9.63	0.008**			
	Color interference time (s)	3.57 ± 5.60	9.93 ± 7.10	9.64 ± 10.06	2.96	0.064	0.132	0.000	0.306
	Word interference time (s)	17.50 ± 5.75	27.00 ± 10.29 #	28.86 ± 14.33 #	6.86	0.005**	0.188	0.006	0.367
	Working memory capacity (score)								
	Immediate detail memory	14.00 ± 4.51	9.71 ± 3.32 #	11.86 ± 4.26	3.90	0.029*	0.167	0.000	0.344
	Delayed detail memory	11.71 ± 4.50	8.57 ± 3.63	7.29 ± 1.68 #	6.02	0.008**	0.236	0.027	0.414
	Immediate structural memory	3.14 ± 1.29	1.93 ± 1.00 #	2.07 ± 1.07 #	4.85	0.013*	0.199	0.010	0.378
	Delayed structural memory	2.21 ± 1.05	1.36 ± 0.84 #	1.21 ± 0.98 #	4.45	0.018*	0.186	0.005	0.364
	Cognitive flexibility capacity								
	Digit connection time (s)	64.00 ± 13.34	73.21 ± 18.32	67.71 ± 41.69	0.40	0.672	0.020	0.000	0.130
	Part A error count	0.21 ± 0.43	0.29 ± 0.47	0.50 ± 0.52	2.72	0.257			
	Digit-alphabet linking time (s)	124.21 ± 40.30	249.57 ± 71.38 #	249.50 ± 70.50 #	25.84	<0.000***	0.491	0.237	0.628
	Part B error count	6.93 ± 3.29	7.36 ± 3.41	6.36 ± 3.80	0.01	0.996			
Visual perceptual ability (score (Secondary Outcomes)	Visual-motor integration (VMI)	108.57 ± 9.48	89.50 ± 5.36 #	84.57 ± 13.20 #	24.04	<0.000***	0.542	0.295	0.667
	Eye-hand coordination	12.21 ± 1.31	9.36 ± 1.99 #	7.93 ± 3.13 #	17.07	<0.000v	0.399	0.144	0.556
	Copying ability	10.50 ± 3.08	7.43 ± 1.56 #	6.79 ± 2.01#	10.38	<0.000***	0.347	0.100	0.513
	Motor-reduced visual perception (MRP)	105.5 ± 9.17	88.57 ± 8.62 #	81.71 ± 7.28 #	29.98	<0.000***	0.606	0.375	0.715
	Figure-ground	11.07 ± 1.27	7.14 ± 1.51 #	6.93 ± 1.90 #	30.44	<0.000***	0.610	0.380	0.718
	Visual closure	12.36 ± 2.65	10.43 ± 2.28	8.79 ± 1.89 #	8.51	0.001**	0.304	0.068	0.475
	Form consistency	8.71 ± 2.92	6.21 ± 3.12	5.00 ± 3.11 #	5.39	0.009**	0.217	0.017	0.395
	General visual perception (GVP)	107.43 ± 7.05	89.57 ± 5.89 #	83.43 ± 9.02 #	39.40	<0.000***	0.669	0.461	0.761

All values are presented as means ± standard deviation (SD). *TD *Typically developing children, *ADHD+DD *Children with attention-deficit/hyperactivity disorder and developmental dyslexia, *CSE *Closed-skill exercise, *OSE *Open- skill exercise. For normally distributed variables, one-way analysis of variance (ANOVA) was used; for variables violating normality or homogeneity of variance, Welch’s ANOVA or Kruskal-Wallis H test was applied. η², partial eta squared (effect size); CI, confidence interval. *p < 0.05, **p < 0.01, ***p < 0.001 (three-group comparison); #p < 0.05 vs. TD group in post hoc pairwise comparisons

### Comparative results of executive function (primary outcomes) in children before and after exercise intervention

Inhibitory Control Outcomes (Tables 4 and 3). Significant Group × Time interactions were found for Stroop B RT, Stroop D RT, Stroop D error count, and word interference time (corrected *p* < 0.05). ADHD + DD groups (OSE and CSE) showed improvements on these measures post-intervention, whereas the TD CSE group only improved on Stroop D RT and word interference time. Post hoc Bonferroni tests showed that the TD CSE group differed significantly from the ADHD + DD CSE group on Stroop B RT (95% CI: -13.261 to -2.953, *p* = 0.001), Stroop D error count (95% CI: -2.023 to -0.048, *p* = 0.037), and word interference time (95% CI: -19.889 to -1.968, *p* = 0.012), but was comparable to the ADHD + DD OSE group on these measures. Both ADHD CSE group (95% CI: -27.553 to -7.590, *p* < 0.001) and ADHD OSE group (95% CI: -20.946 to − 0.983, *p* = 0.027) differed significantly from the TD group on Stroop D RT. ANCOVA with baseline as covariate revealed significant group differences in Stroop B RT, Stroop D RT, and word interference time. Post hoc tests showed significant differences between the ADHD OSE and ADHD CSE groups: Stroop B RT (95% CI: -7.297 to -0.039, *p* = 0.047), Stroop D RT (95% CI: -18.535 to -8.913, *p* < 0.001), and word interference time (95% CI: -17.196 to -4.692, *p* < 0.001). A significant difference in Stroop D RT was also observed between the ADHD OSE group and the TD CSE group (95% CI: -17.028 to -5.369, *p* < 0.001).

**Table 3:** Descriptive statistics and repeated-measures ANOVA results for primary outcome measures across three groups at pre-intervention and post-intervention

Variables	Groups						ANOVA									
	TD (CSE)		ADHD+DD (CSE)		ADHD+DD (OSE)		Time			Group			Group × Time			
	PRE	POST	PRE	POST	PRE	POST	F	p	ηₚ²	F	p	ηₚ²	F	p	ηₚ²	FDR-corrected†
Inhibitory control capacity																
Stroop A RT (s)	19.14 ± 3.39	19.43 ± 3.63	25.43 ± 7.21	24.00 ± 6.40	23.86 ± 2.93	20.57 ± 2.77	5.692	0.022*	0.127	5.653	0.007**	0.225	2.778	0.075	0.125	-
Stroop A error count	0.07 ± 0.27	0.14 ± 0.36	0.21 ± 0.43	0.14 ± 0.36	0.21 ± 0.43	0.07 ± 0.27	0.667	0.419	0.017	0.193	0.825	0.010	1.167	0.322	0.056	-
Stroop B RT (s)	24.57 ± 4.07	25.5 ± 5.07	34.29 ± 7.16	32.00 ± 7.46	32.07 ± 6.74	26.86 ± 3.30	11.067	0.002 **	0.221	7.765	0.001**	0.285	7.258	0.002**	0.271	†
Stroop B error count	0.14 ± 0.36	0.14 ± 0.36	0.86 ± 1.03	0.71 ± 0.83	0.86 ± 0.86	0.64 ± 0.75	2.876	0.098	0.069	3.665	0.035*	0.158	0.805	0.454	0.040	-
Stroop C RT (s)	23.07 ± 6.03	23.43 ± 3.20	35.64 ± 12.29	33.71 ± 11.4	33.50 ± 10.42	30.93 ± 8.83	4.353	0.044*	0.100	6.246	0.004**	0.243	1.803	0.178	0.085	-
Stroop C error count	0.29 ± 0.47	0.36 ± 0.63	1.14 ± 1.29	0.86 ± 0.95	1.07 ± 1.07	0.50 ± 0.65	5.848	0.020*	0.130	2.511	0.094	0.114	2.948	0.064	0.131	-
Stroop D RT (s)	42.36 ± 7.36	39.93 ± 6.34	60.64 ± 13.56	56.79 ± 14.45	60.93 ± 12.60	43.29 ± 8.37	85.252	0.000***	0.686	9.896	0.000***	0.337	31.533	0.000***	0.618	†
Stroop D error count	1.00 ± 1.18	1.14 ± 0.77	2.36 ± 1.15	1.86 ± 1.03	2.64 ± 1.65	1.36 ± 1.01	12.258	0.001**	0.239	4.166	0.023*	0.176	6.975	0.003**	0.263	†
Color interference time (s)	3.57 ± 5.60	3.64 ± 5.18	9.93 ± 7.10	9.21 ± 6.18	9.64 ± 10.06	10.36 ± 8.69	0.001	0.977	0.000	3.832	0.030*	0.164	0.247	0.782	0.013	-
Word interference time (s)	17.50 ± 5.75	13.79 ± 8.18	27.00 ± 10.29	26.14 ± 12.43	28.86 ± 14.33	16.43 ± 7.49	24.351	0.000***	0.384	4.777	0.014*	0.197	9.184	0.001**	0.320	†
Working memory capacity (score)																
Immediate detail memory	14.00 ± 4.51	18.57 ± 5.52	9.71 ± 3.32	18.86 ± 4.13	11.86 ± 4.26	19. 00 ± 3.31	116.628	0.000***	0.749	1.035	0.365	0.050	4.224	0.022*	0.178	†
Delayed detail memory	11.71 ± 4.50	15.00 ± 5.13	8.57 ± 3.63	16.43 ± 5.45	7.29 ± 1.68	17.43 ± 3.46	150.398	0.000***	0.794	0.296	0.746	0.015	12.140	0.000***	0.384	†
Immediatestructural memory	3.14 ± 1.29	3.50 ± 1.02	1.93 ± 1.00	3.00 ± 1.30	2.07 ± 1.07	3.36 ± 0.63	27.364	0.000***	0.412	3.225	0.051	0.142	2.634	0.085	0.119	-
Delayedstructural memory	2.21 ± 1.05	2.64 ± 1.01	1.36 ± 0.84	2.43 ± 1.16	1.21 ± 0.98	3.00 ± 0.56	73.551	0.000***	0.653	1.388	0.262	0.066	9.420	0.000***	0.326	†
Cognitive flexibility capacity																
Digit connection time (s)	64.00 ± 13.34	52.14 ± 13.72	73.21 ± 18.32	70.43 ± 28.76	67.71 ± 41.69	64.00 ± 13.34	4.025	0.052	0.094	1.205	0.311	0.058	0.458	0.636	0.023	-
Part A error count	0.21 ± 0.43	0.21 ± 0.43	0.29 ± 0.47	0.29 ± 0.47	0.50 ± 0.52	0.21 ± 0.43	0.411	0.525	0.010	0.700	0.503	0.035	0.411	0.666	0.021	-
Digit-alphabet linking time (s)	124.21 ± 40.30	112.57 ± 35.61	249.57 ± 71.38	247.21 ± 75.84	249.50 ± 70.50	124.21 ± 40.30	13.015	0.001**	0.250	26.576	0.000***	0.577	8.528	0.001**	0.304	†
Part B error count	6.93 ± 3.29	5.50 ± 3.21	7.36 ± 3.41	5.71 ± 2.34	6.36 ± 3.80	6.93 ± 3.29	8.636	0.006**	0.181	1.356	0.270	0.065	0.268	0.766	0.014	-

All values are presented as means ± standard deviation (SD). *TD *Typically developing children, *ADHD+DD *Children with attention-deficit/hyperactivity disorder and developmental dyslexia, *CSE *Closed-skill exercise, *OSE *Open- skill exercise, *PRE *Pre-intervention, *POST *Post-intervention; ηp², partial eta-squared effect size. *p < 0.05, **p < 0.01, ***p < 0.001 (three-group comparison). † Remained significant after FDR correction (Benjamini-Hochberg, q = 0.05) for Time × Group interactions of primary outcomes. “-” not significant after FDR correction. For secondary outcomes (visual perception), results are exploratory with unadjusted p-values

**Table 4:** Descriptive statistics and repeated-measures ANOVA results for secondary outcome measures across three groups at pre-intervention and post-intervention

Variables	Groups						ANOVA								
	TD (CSE)		ADHD+DD (CSE)		ADHD+DD (OSE)		Time			Group			Group × Time		
	PRE	POST	PRE	POST	PRE	POST	F	p	ηₚ²	F	p	ηₚ²	F	p	ηₚ²
VMI	108.57 ± 9.48	103.43 ± 14.55	89.50 ± 5.36	89.71 ± 10.03	84.57 ± 13.20	107.07 ± 21.46	6.043	0.019*	0.134	8.126	0.001**	0.294	12.618	0.000***	0.393
Eye-hand coordination	12.21 ± 1.31	12.79 ± 2.16	9.36 ± 1.99	10.21 ± 1.58	7.93 ± 3.13	11.07 ± 3.43	12.812	0.001**	0.247	10.056	0.000***	0.340	3.654	0.035*	0.158
Copying ability	10.50 ± 3.08	9.07 ± 2.76	7.43 ± 1.56	6.00 ± 2.00	6.79 ± 2.01	11.29 ± 4.20	1.258	0.269	0.031	7.145	0.002**	0.268	16.387	0.000***	0.457
MRP	105.57 ± 9.17	106.21 ± 8.77	88.57 ± 8.62	99.29 ± 15.98	81.71 ± 7.28	112.71 ± 10.40	42.683	0.000***	0.523	8.976	0.001**	0.315	17.064	0.000***	0.467
Figure-ground	11.07 ± 1.27	11.21 ± 2.42	7.14 ± 1.51	9.07 ± 3.27	6.93 ± 1.90	11.00 ± 1.92	21.357	0.000***	0.354	13.369	0.000***	0.407	6.569	0.003**	0.252
Visual closure	12.36 ± 2.65	12.79 ± 2.49	10.43 ± 2.28	11.79 ± 3.19	8.79 ± 1.89	13.57 ± 2.95	20.414	0.000***	0.344	2.198	0.125	0.101	7.470	0.002**	0.277
Form consistency	8.71 ± 2.92	9.21 ± 2.01	6.21 ± 3.12	8.07 ± 3.39	5.00 ± 3.11	11.50 ± 2.44	21.544	0.000***	0.356	2.961	0.064	0.132	8.156	0.001**	0.295
GVP	107.43 ± 7.05	105.29 ± 8.34	89.57 ± 5.89	95.43 ± 11.51	83.43 ± 9.02	110.93 ± 12.71	36.571	0.000***	0.484	12.170	0.000***	0.384	26.482	0.000***	0.576

All values are presented as means ± standard deviation (SD). *TD *Typically developing children, *ADHD+DD *Children with attention-deficit/hyperactivity disorder and developmental dyslexia, *CSE *Closed-skill exercise, *OSE *Open- skill exercise, *VMI *Visual-motor integration, *MRP *Motor-reduced visual perception, *GVP *General visual perception, *PRE *Pre-intervention, *POST *Post-intervention; ηp², partial eta-squared effect size. *p < 0.05, **p < 0.01, ***p < 0.001 (three-group comparison)

Working Memory Outcomes (Tables 4 and 3). Significant Group × Time interactions were observed for immediate detail memory, delayed detail memory, and delayed structural memory (corrected *p* < 0.05). For immediate detail memory, no significant post-intervention group differences were found among the three groups (all *p* > 0.05), while a significant main effect of time was evident (95% CI: -8.255 to -5.650, *p* < 0.001), indicating general improvements across all groups. For delayed detail memory, similarly, there were no significant group differences (all *p* > 0.05), whereas the main effect of time remained significant (95% CI: -8.265 to -5.925, *p* < 0.001). Notably, ANCOVA revealed significant group differences after baseline adjustment, with both the ADHD CSE group (95% CI: 0.300 to 7.932, *p* = 0.031) and the ADHD OSE group (95% CI: 2.166 to 10.265, *p* = 0.001) performing significantly better than the TD CSE group. For delayed structural memory, there were also showed no significant group differences (all *p* > 0.05), with a significant main effect of time observed (95% CI: -1.354 to -0.837, *p* < 0.001). Further ANCOVA results indicated that the ADHD OSE group achieved significantly better performance than the TD CSE group (95% CI: 0.194 to 1.741, *p* = 0.01), while the difference between the ADHD OSE and ADHD CSE groups approached statistical significance (95% CI: -0.051 to 1.368, *p* = 0.077).

Cognitive Flexibility Outcomes (Tables 4 and 3). Significant Group × Time interaction was observed for the digit–alphabet linking time task (corrected *p* < 0.05). Post hoc Bonferroni comparisons indicated significant differences in the ADHD + DD OSE group and the TD CSE group (95% CI: 38.728 to 129.200, *p* < 0.001), as well as between the ADHD + DD OSE group and the ADHD + DD CSE group (95% CI: -91.272 to -0.800, *p* = 0.045). A significant difference was also found between the ADHD + DD CSE group and the TD CSE group (95% CI: 84.764 to 175.236, *p* < 0.001). ANCOVA revealed significant group differences in the digit–alphabet linking time task, primarily between the ADHD OSE and ADHD CSE groups (95% CI: -138.866 to -45.088, *p* < 0.001) and between the TD CSE and ADHD CSE groups (95% CI: -156.383 to -33.254, *p* = 0.001).

### Comparative results of visual perceptual abilities (secondary outcomes) in children before and after exercise intervention

Secondary outcome measures (VMI, eye-hand coordination, copying ability, MRP, figure-ground, visual closure, form consistency, and GVP) were analyzed using linear mixed models (Table 5), with significant Group × Time interactions observed across all indices (all *p* < 0.05). After adjusting for baseline performance via ANCOVA (Table 3), significant post-intervention group differences remained for VMI, copying ability, MRP, form consistency, and GVP (all *p* < 0.05), whereas no significant adjusted differences were found for eye-hand coordination, figure-ground, or visual closure.

**Table 5:** ANCOVA results comparing post-intervention outcome measures across three groups, adjusted for pre-intervention baseline values

Variables	Groups									ANCOVA		
	TD CSE			ADHD+DD CSE			ADHD+DD OSE					
	PRE	POST	Δ	PRE	POST	Δ	PRE	POST	Δ	F	p	ηₚ²
Stroop A RT (s)	19.14 ± 3.39	19.43 ± 3.63	0.29 ± 4.70	25.43 ± 7.21	24.00 ± 6.40	-1.43 ± 1.60	23.86 ± 2.93	20.57 ± 2.77	-3.29 ± 4.86	1.814	0.177	0.087
Stroop A error count	0.07 ± 0.27	0.14 ± 0.36	0.07 ± 0.48	0.21 ± 0.43	0.14 ± 0.36	-0.07 ± 0.27	0.21 ± 0.43	0.07 ± 0.27	-0.14 ± 0.36	0.609	0.549	0.031
Stroop B RT (s)	24.57 ± 4.07	25.50 ± 5.07	0.93 ± 4.25	34.29 ± 7.16	32.00 ± 7.46	-2.29 ± 2.27	32.07 ± 6.74	26.86 ± 3.30	-5.21 ± 5.61	4.213	0.022**	0.182
Stroop B error count	0.14 ± 0.36	0.14 ± 0.36	0.00 ± 0.56	0.86 ± 1.03	0.71 ± 0.83	-0.14 ± 0.36	0.86 ± 0.86	0.64 ± 0.75	-0.21 ± 0.43	0.162	0.851	0.008
Stroop C RT (s)	23.07 ± 6.03	23.43 ± 3.20	0.36 ± 5.90	35.64 ± 12.29	33.71 ± 11.40	-1.93 ± 2.46	33.5 ± 10.42	30.93 ± 8.83	-2.57 ± 3.78	0.320	0.728	0.017
Stroop C error count	0.29 ± 0.47	0.36 ± 0.63	0.07 ± 0.62	1.14 ± 1.29	0.86 ± 0.95	-0.29 ± 0.73	1.07 ± 1.07	0.50 ± 0.65	-0.57 ± 0.76	1.462	0.244	0.071
Stroop D RT (s)	42.36 ± 7.36	39.93 ± 6.34	-2.43 ± 4.07	60.64 ± 13.56	56.79 ± 14.45	-3.86 ± 4.06	60.93 ± 12.60	43.29 ± 8.37	-17.64 ± 7.81	27.535	0.000***	0.592
Stroop D error count	1.00 ± 1.18	1.14 ± 0.77	0.14 ± 1.17	2.36 ± 1.15	1.86 ± 1.03	-0.50 ± 0.65	2.64 ± 1.65	1.36 ± 1.01	-1.29 ± 1.14	2.963	0.064	0.135
Color interference time (s)	3.57 ± 5.60	3.64 ± 5.18	0.07 ± 6.73	9.93 ± 7.10	9.21 ± 6.18	-0.71 ± 2.67	9.64 ± 10.06	10.36 ± 8.69	0.71 ± 5.88	1.122	0.336	0.056
Word interference time (s)	17.50 ± 5.75	13.79 ± 8.18	-3.71 ± 3.91	27.00 ± 10.29	26.14 ± 12.43	-0.86 ± 5.45	28.86 ± 14.33	16.43 ± 7.49	-12.43 ± 11.01	9.685	0.000***	0.338
Immediate detail memory	14.00 ± 4.51	18.57 ± 5.52	4.57 ± 4.67	9.71 ± 3.32	18.86 ± 4.13	9.14 ± 4.42	11.86 ± 4.26	19.00 ± 3.31	7.14 ± 3.30	1.476	0.241	0.072
Delayed detail memory	11.71 ± 4.50	15.00 ± 5.13	3.29 ± 2.79	8.57 ± 3.63	16.43 ± 5.45	7.86 ± 5.11	7.29 ± 1.68	17.43 ± 3.46	10.14 ± 2.88	7.555	0.002***	0.285
Immediate structural memory	3.14 ± 1.29	3.50 ± 1.02	0.36 ± 0.75	1.93 ± 1.00	3.00 ± 1.30	1.07 ± 1.49	2.07 ± 1.07	3.36 ± 0.63	1.29 ± 0.99	0.481	0.622	0.025
Delayed structural memory	2.21 ± 1.05	2.64 ± 1.01	0.43 ± 0.51	1.36 ± 0.84	2.43 ± 1.16	1.07 ± 1.07	1.21 ± 0.98	3.00 ± 0.56	1.79 ± 0.80	5.335	0.009***	0.219
Digit connection time (s)	64.00 ± 13.34	52.14 ± 13.72	-11.86 ± 7.53	73.21 ± 18.32	70.43 ± 28.76	-2.79 ± 20.56	67.71 ± 41.69	57.07 ± 35.96	-10.64 ± 41.77	1.186	0.317	0.059
Part A error count	0.21 ± 0.43	0.21 ± 0.43	0.00 ± 0.68	0.29 ± 0.47	0.29 ± 0.47	0.00 ± 0.68	0.50 ± 0.52	0.29 ± 0.83	-0.21 ± 0.80	0.036	0.965	0.002
Digit-alphabet linking time (s)	124.21 ± 40.30	112.57 ± 35.61	-11.64 ± 10.42	249.57 ± 71.38	247.21 ± 75.84	-2.36 ± 79.63	249.50 ± 70.50	155.21 ± 36.50	-94.29 ± 78.51	14.374	0.000***	0.431
Part B error count	6.93 ± 3.29	5.50 ± 3.21	-1.43 ± 3.50	7.36 ± 3.41	5.71 ± 2.34	-1.64 ± 4.50	6.36 ± 3.80	3.86 ± 2.71	-2.50 ± 4.22	1.639	0.208	0.079
VMI	108.57 ± 9.48	103.43 ± 14.55	-5.14 ± 14.34	89.50 ± 5.36	89.71 ± 10.03	0.21 ± 11.97	84.57 ± 13.20	107.07 ± 21.46	22.50 ± 19.15	6.205	0.005**	0.246
Eye-hand coordination	12.21 ± 1.31	12.79 ± 2.16	0.57 ± 1.99	9.36 ± 1.99	10.21 ± 1.58	0.86 ± 2.32	7.93 ± 3.13	11.07 ± 3.43	3.14 ± 3.68	1.671	0.202	0.081
Copying ability	10.50 ± 3.08	9.07 ± 2.76	-1.43 ± 3.32	7.43 ± 1.56	6.00 ± 2.00	-1.43 ± 1.87	6.79 ± 2.01	11.29 ± 4.20	4.50 ± 3.94	12.519	0.000***	0.397
MRP	105.57 ± 9.17	106.21 ± 8.77	0.64 ± 7.36	88.57 ± 8.62	99.29 ± 15.98	10.71 ± 20.14	81.71 ± 7.28	112.71 ± 10.40	31.00 ± 11.34	4.425	0.019*	0.189
Figure-ground	11.07 ± 1.27	11.21 ± 2.42	0.14 ± 2.28	7.14 ± 1.51	9.07 ± 3.27	1.93 ± 3.61	6.93 ± 1.90	11.00 ± 1.92	4.07 ± 2.56	2.051	0.143	0.097
Visual closure	12.36 ± 2.65	12.79 ± 2.49	0.43 ± 3.20	10.43 ± 2.28	11.79 ± 3.19	1.36 ± 3.75	8.79 ± 1.89	13.57 ± 2.95	4.79 ± 2.29	2.412	0.103	0.113
Form consistency	8.71 ± 2.92	9.21 ± 2.01	0.50 ± 2.14	6.21 ± 3.12	8.07 ± 3.39	1.86 ± 4.91	5.00 ± 3.11	11.50 ± 2.44	6.50 ± 4.72	5.379	0.009**	0.221
GVP	107.43 ± 7.05	105.29 ± 8.34	-2.14 ± 8.52	89.57 ± 5.89	95.43 ± 11.51	5.86 ± 14.83	83.43 ± 9.02	110.93 ± 12.71	27.50 ± 8.97	9.454	0.000***	0.332

All values are presented as means ± standard deviation (SD). *TD *Typically developing children; ADHD+DD, children with attention-deficit/hyperactivity disorder and developmental dyslexia, *CSE *Closed-skill exercise, *OSE *Open- skill exercise, *VMI *Visual-motor integration, *MRP *Motor-reduced visual perception, *GVP *General visual perception, *PRE *Pre-intervention, *POST *Post-intervention; Δ, change score (POST − PRE); ηp², partial eta-squared effect size. *p < 0.05, **p < 0.01, ***p < 0.001 (three-group comparison)

## Discussion

This study aimed to explore the effects of OSE and CSE interventions on EF (including inhibitory control, working memory, and cognitive flexibility) and visual perceptual abilities in children with ADHD + DD. TD children were recruited as the baseline control group and received CSE training. The results showed that exercise intervention yielded universal benefits for children’s cognitive development, with all participants exhibiting varying degrees of improvement in the above indicators. There were distinct differences in the effects between the two exercise modes: OSE demonstrated significant advantages in improving inhibitory control and cognitive flexibility. CSE only produced limited intervention effects. By contrast, OSE could effectively narrow the developmental gap between children with ADHD + DD and their TD peers, presenting superior overall intervention efficacy relative to CSE. Notably, the improvements in various dimensions of visual perception induced by different exercise interventions in this study are only exploratory findings, and the specific pathways and underlying mechanisms remain to be further verified in subsequent empirical studies.

This research confirms that OSE (table tennis training) significantly improves EF in children with ADHD + DD through its unique dynamic interactive patterns. The specific manifestations are as follows: (1) Inhibitory control was markedly improved in OSE relative to CSE, which only yielded limited intervention effects, as the gains stemmed from the demands of response inhibition when confronting unpredictable ball trajectories during OSE; (2) For working memory, all groups improved after exercise intervention, with OSE producing more prominent compensatory gains in delayed memory among children with ADHD + DD, owing to its demand for real-time encoding of movement sequences and continuous adaptation to variations in ball speed and spin [22]; (3) Cognitive flexibility was enhanced primarily due to the tactical adjustment demands during multi-rally exchanges, and relevant studies have verified that interactive cognitive-motor interventions can effectively ameliorate core cognitive symptoms in school-age children with ADHD [23]. Such precise neuromuscular regulation based on real-time environmental feedback effectively strengthens the dynamic integration function of the prefrontal-basal ganglia circuit [24].

The 12-week OSE (table tennis training)—emphasizing multi-dimensional environmental adaptation, including multi-ball tracking requiring prediction of random trajectories, visual gestalt tasks integrated with dynamic background interference, and spatial training with unexpected landing position changes—may potentially enhance visual perception through three plausible mechanisms: (1) OSE service reception training, which requires coping with unpredictable ball paths, may strengthen the fine-tuning ability of eye-hand coordination; (2) Processing of variable-speed and variable-spin balls could improve dynamic figure-ground segregation; (3) Real-time trajectory prediction in three-dimensional space may enhance visual tracking adaptability. These perceptual improvements may speculatively reflect neuroplastic characteristics associated with OSE: continuous processing of environmental uncertainty [25, 26] might enhance the timeliness of multisensory integration [27–29], and potentially facilitate coordinated activation of the frontoparietal attention network and the cerebellar prediction system [30–32], which could contribute to the formation of more flexible visuospatial processing pathways. It should be noted that the above neural mechanism interpretations remain exploratory and need further empirical verification.

In CSE (track-and-field training), fixed-pattern running, jumping and throwing tasks (e.g., timed and distance-controlled running, standardized shot put) appear to show relatively limited capacity to effectively activate the dorsolateral prefrontal cortex closely linked to working memory, largely due to the absence of dynamic real-time information encoding demands inherent to OSE. Its preset movement sequences (e.g., fixed start-acceleration-sprint routines) cannot fully simulate the millisecond-level response inhibition demands required in table tennis for coping with unexpected ball trajectories, which may lead to relatively lower activation of the anterior cingulate cortex. Furthermore, the relatively stable and fixed environment of track-and-field CSE reduces the necessity of real-time tactical adjustment. The magnitude of improvement in WCST correct response number in the CSE group was significantly lower than that in the OSE group, which may preliminarily imply insufficient plastic induction of the prefrontal-striatal circuit rather than definitive causal confirmation.

Built on empirical behavioral data, the present movement intervention model may provide a feasible reference for inclusive education and supportive services for children with special needs [33], and is consistent with the developmental orientation of China’s Education Modernization 2035 in promoting educational equity and quality improvement. This study preliminarily explored the effects of early structured movement intervention on cognitive-perceptual development among children with neurodevelopmental disorders, with observable benefits in executive function and visual perception. The findings may offer practical implications for improving their social participation and school adaptation and help preliminarily clarify the potential association between motor intervention and developmental support.

Limitations. Despite confirming OSE (table tennis training) effectively improves EF and visual perception in ADHD + DD children via rigorous controlled trials, limitations remain. First, TD children only received CSE, with no TD OSE control group. Hence, the cognitive improvements of ADHD + DD children in the OSE group cannot be solely attributed to exercise type, since novelty and general cognitive engagement may also account for the gains. Second, this study lacked a non-intervention ADHD + DD control group, so slight improvements in EF and visual perception could not be ruled out due to regular schooling and natural developmental maturation. Third, although participants were children with ADHD + DD, all assessments only examined EF and visual perception. No direct measures of reading ability or reading-related deficits were included and the core pathological characteristics of children with ADHD + DD were not fully incorporated for comprehensive assessment. Fourth, this study did not explore exercise dose–response relationships or long-term follow-up of intervention effects. In addition, this study did not conduct equivalence testing to strictly verify whether the intervention effect could bring the performance of ADHD + DD children close to the level of TD children; relevant phenotypic improvement results are only exploratory rather than definitive normalization conclusions. Meanwhile, no in-depth neural mechanism examinations such as electroencephalography (EEG) or neuroimaging were conducted. Only external behavioral phenotypic changes could be observed, and the intrinsic neural pathways and functional targets underlying the intervention effects could not be revealed from the perspectives of brain region activation and neural circuit plasticity.

## Conclusions

The findings of this study indicate that, compared with CSE ( track-and-field training), OSE ( table tennis training) yields more pronounced improvements in EF and visual perception among children with ADHD + DD. After a 12-week structured intervention program, participants in the OSE group showed significant behavioral gains in EF and visual perception. However, as no equivalence testing was performed in the present study, we cannot confirm that the intervention normalized their performance to the level of typically developing peers. These results provide preliminary empirical evidence supporting structured OSE as a promising non-pharmacological means to improve EF and visual perception deficits in children with ADHD + DD.

## Supplementary Information

Supplementary Material 1.

Supplementary Material 2.

## Data Availability

The dataset supporting the conclusions of this article is included in the article.

## Abbreviations

ADHD+DD: Attention Deficit/Hyperactivity Disorder and Comorbid Developmental Dyslexia
OSE: Open-skill exercise
CSE: Closed-skill exercise
EF: Executive Function
TD: Typically Developing
SCWT: Stroop Color Word Test
CFT: Complex Figure Test
TMT: Trail Making Test
DTVP-3: Developmental Test of Visual Perception, Third Edition
VMI: Visual-Motor Integration
MRP: Motor Reduced Visual Perception
GVP: General Visual Perception
ANOVA: Analysis of Variance
LD: Learning Disability
CBT: Cognitive Behavioral Therapy
DSM-V: Diagnostic and Statistical Manual of Mental Disorders, Fifth Edition
WISC: Wechsler Intelligence Scale for Children
RT: Response Time
EEG: Electroencephalography
