# Supplementary material for: Comparative effectiveness of 12-week open- and closed-skill exercises on executive function in children with ADHD and developmental dyslexia: a randomized controlled trial
Source: BMC Pediatr. 2026 Jun 15;26:775. doi: 10.1186/s12887-026-07105-w (PMC13501822; doi:10.1186/s12887-026-07105-w)
Supplement: Supplementary file 1 [file 12887_2026_7105_MOESM2_ESM.docx]

Highlights

- ADHD+DD children showed EF and visual perception impairments compared to TD peers.
- OSE and CSE interventions improved EF and visual perception in ADHD+DD children.
- OSE interventions demonstrated superior efficacy compared to CSE interventions.
